# ‘Raisin bread sign’ feature of pontine autosomal dominant microangiopathy and leukoencephalopathy

**DOI:** 10.1093/braincomms/fcad281

**Published:** 2023-10-22

**Authors:** Mai Kikumoto, Takashi Kurashige, Tomohiko Ohshita, Kodai Kume, Osamu Kikumoto, Tomohisa Nezu, Shiro Aoki, Kazuhide Ochi, Hiroyuki Morino, Eiichi Nomura, Hiroshi Yamashita, Mayumi Kaneko, Hirofumi Maruyama, Hideshi Kawakami

**Affiliations:** Department of Clinical Neuroscience and Therapeutics, Hiroshima University Graduate School of Biomedical and Health Sciences, Hiroshima 7348551, Japan; Department of Neurology, Hiroshima City North Medical Center Asa Citizens Hospital, Hiroshima 7310293, Japan; Department of Molecular Epidemiology, Research Institute for Radiation Biology and Medicine, Hiroshima University, Hiroshima 7348553, Japan; Department of Neurology, National Hospital Organization Kure Medical Center and Chugoku Cancer Center, Kure 7370023, Japan; Department of Clinical Neuroscience and Therapeutics, Hiroshima University Graduate School of Biomedical and Health Sciences, Hiroshima 7348551, Japan; Department of Neurology, Hiroshima City North Medical Center Asa Citizens Hospital, Hiroshima 7310293, Japan; Department of Neurology, National Hospital Organization Kure Medical Center and Chugoku Cancer Center, Kure 7370023, Japan; Department of Molecular Epidemiology, Research Institute for Radiation Biology and Medicine, Hiroshima University, Hiroshima 7348553, Japan; Ideshita Clinic, Hiroshima 7391734, Japan; Department of Clinical Neuroscience and Therapeutics, Hiroshima University Graduate School of Biomedical and Health Sciences, Hiroshima 7348551, Japan; Department of Clinical Neuroscience and Therapeutics, Hiroshima University Graduate School of Biomedical and Health Sciences, Hiroshima 7348551, Japan; Department of Neurology, Hiroshima City North Medical Center Asa Citizens Hospital, Hiroshima 7310293, Japan; Department of Neurology, Hiroshima Prefectural Hospital, Hiroshima 7348530, Japan; Department of Clinical Neuroscience and Therapeutics, Hiroshima University Graduate School of Biomedical and Health Sciences, Hiroshima 7348551, Japan; Department of Medical Genetics, Tokushima University Graduate School of Biomedical Sciences, Tokushima 7708503, Japan; Department of Neurology, Hiroshima City Hiroshima Citizens Hospital, Hiroshima 7308518, Japan; Department of Neurology, Hiroshima City North Medical Center Asa Citizens Hospital, Hiroshima 7310293, Japan; Department of Diagnostic Pathology, Hiroshima City North Medical Center Asa Citizens Hospital, Hiroshima 7310293, Japan; Department of Clinical Neuroscience and Therapeutics, Hiroshima University Graduate School of Biomedical and Health Sciences, Hiroshima 7348551, Japan; Department of Molecular Epidemiology, Research Institute for Radiation Biology and Medicine, Hiroshima University, Hiroshima 7348553, Japan

**Keywords:** PADMAL, *COL4A1*, hereditary stroke, MRI, cerebral small vessel disease and pons

## Abstract

Pontine autosomal dominant microangiopathy and leukoencephalopathy is one of hereditary cerebral small vessel diseases caused by pathogenic variants in *COL4A1* 3′UTR and characterized by multiple small infarctions in the pons. We attempted to establish radiological features of this disease. We performed whole exome sequencing and Sanger sequencing in one family with undetermined familial small vessel disease, followed by clinicoradiological assessment and a postmortem examination. We subsequently investigated clinicoradiological features of patients in a juvenile cerebral vessel disease cohort and searched for radiological features similar to those found in the aforementioned family. Sanger sequencing was performed in selected cohort patients in order to detect variants in the same gene. An identical variant in the *COL4A1* 3′UTR was observed in two patients with familial small vessel disease and the two selected patients, thereby confirming the pontine autosomal dominant microangiopathy and leukoencephalopathy diagnosis. Furthermore, postmortem examination showed that the distribution of thickened media tunica and hyalinized vessels was different from that in lacunar infarctions. The appearance of characteristic multiple oval small infarctions in the pons, which resemble raisin bread, enable us to make a diagnosis of pontine autosomal dominant microangiopathy and leukoencephalopathy. This feature, for which we coined the name ‘raisin bread sign’, was also correlated to the pathological changes.

## Teaser

Kikumoto *et al*. detected a characteristic radiological feature of pontine autosomal dominant angiopathy and leukoencephalopathy, which enables more accurate evaluations of patients with undetermined juvenile cerebral vascular disorder. This feature is multiple oval small infarctions in the pons resembling raisin bread, for which they coined the name ‘raisin bread sign’.

## Graphical Abstract

**Graphical Abstract:**
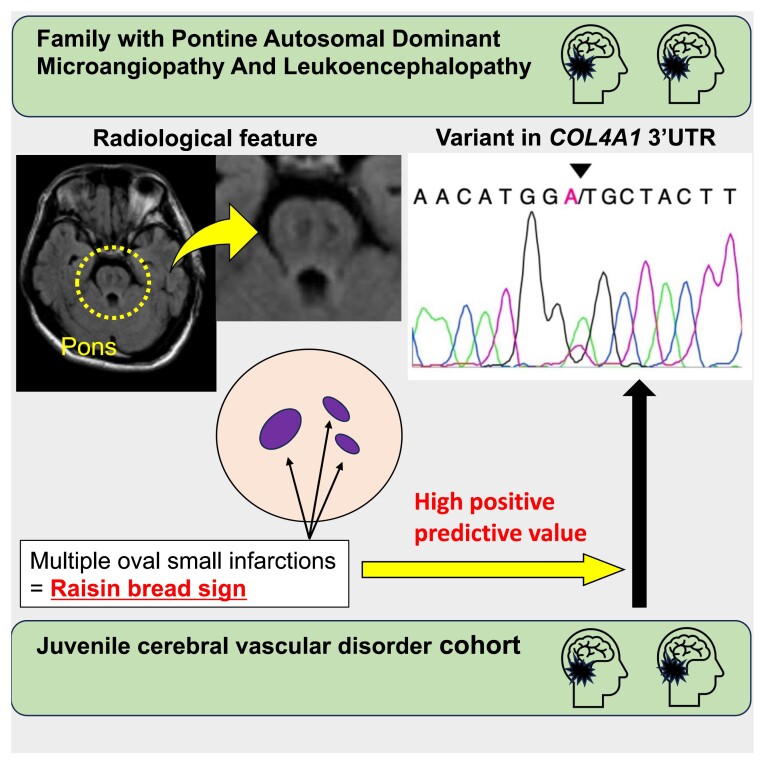

## Introduction

Over the last decades, several monogenic cerebral small vessel diseases (cSVD) have been reported, with the diagnostic value of some of the radiological features having been identified.^1-5^ Pontine autosomal dominant microangiopathy and leukoencephalopathy (PADMAL) is one of the hereditary cSVD that is caused by pathogenic variants in the *COL4A1* 3′ untranslated region (UTR).^6^  *COL4A1* encodes collagen type IV alpha 1 chain. Collagen type IV is one of the components of the brain vascular basement membrane responsible for maintaining mechanical stability.^7^ The causative variants in the *COL4A1* 3′UTR lead to upregulation of collagen type IV.^6^ The recurrent ischaemic episodes of PADMAL are likely to start in patients from their mid-30s to mid-40s, with the radiological feature characterized by pontine multiple small infarctions and leukoencephalopathy.^6^ The neuropathological findings for vessels in the brain and skin tissue show the accumulation of collagen type IV between the endothelial cells and vascular smooth muscle cells.^6,8^ While loss-of-function effect associated with the variant in *COL4A1* can result in the dysfunction of vessels, including intracranial haemorrhage,^9^ the occurrence of haemorrhage in PADMAL is relatively rare.

Although several cases of PADMAL have been reported worldwide, including Asia,^10-12^ key signs for determining a PADMAL diagnosis have yet to be established. As a result, this situation makes it more difficult to diagnose young adult patients with undetermined cSVD as PADMAL. While certain risk factors of PADMAL have not been established yet, sporting activities with a high risk of head trauma or prolonged exercise are considered to be avoided for patients with *COL4A1/2* variants..^5^ Therefore, if we can detect patients with PADMAL more precisely, we would be able to recognize higher risks of recurrent brain infarctions with them. Here, we present important radiological and neuropathological features of patients with PADMAL, which makes it possible to more accurately screen patients with PADMAL.

## Materials and methods

### Participants

First, we clinicoradiologically and genetically examined two patients (F1-IV-2 and F1-IV-6) with undetermined familial cSVD and five unaffected relatives in Family 1. Their family history and hemiparesis enabled us to make a diagnosis of familial cSVD.

Second, we then performed a retrospective cohort study that evaluated clinicoradiological features of the patients with juvenile cerebral vascular disorder (CVD) (*n* = 40; age of the onset between age 31 and 50 years) who were admitted to Hiroshima University Hospital from January 2011 to April 2021. We included consecutive patients who were diagnosed with acute brain infarctions based on the clinical onset and radiological findings within 7 days from the onset.

### Ethical consideration

This study was approved by the Ethics Committees of the participating institutions. Written informed consent was obtained from all participants who underwent genetic analysis or their legal representatives.

### Neuroimaging

We clarified the radiological feature shared by two patients (F1-IV-2 and F1-IV-6) in Family 1 by assessing their findings on MRI. Subsequently, we then screened a cohort of CVD patients and selected patients exhibiting radiological features that were similar to those observed in F1-IV-2 and F1-IV-6. Patients in Family 1 and the CVD cohort were radiologically examined by either or both of CT and MRI. Sequences of MRI included at least diffusion weighted imaging, fluid attenuated inversion recovery, MR angiography and T2*. Two observers (M.K. and T.O.) consistently rated MR images of patients in Family 1 and the juvenile CVD cohort with their clinical information.

### Whole exome sequencing

We performed whole exome sequencing in patients (F1-IV-2 and F1-IV-6) and their unaffected relative (F1-IV-1) in Family 1 using the Illumina platform (Illumina, San Diego, CA). Genomic DNA was extracted from peripheral blood leukocytes by using QuickGene-610L (Kurabo Industries Ltd., Osaka, Japan). Mapping to the human genome reference (GRCh37) was done using the Burrows–Wheeler alignment tool, with the removal of duplicate reads performed by Picard. Variant calls and annotation were done using GATK and Annovar.

### Sanger sequencing

Sanger sequencing was performed by using 3130xl Genetic Analyzer (Thermo Fisher Scientific, Waltham, MA) in F1-III-5, F1-III-8, F1-III-9, F1-IV-1, F1-IV-2, F1-IV-6, F1-IV-7 and the patients selected from the cohort (F2-IV-8 and F3-III-2). Using this procedure, we were able to confirm the variant detected in whole exome sequencing. The forward and reverse primer sequences used in the polymerase chain reaction were 5′-AGGTCAATGAAGCAGGGTGT-3′ and 5′- GCCATGTTTCTGACGTGCTG-3′, respectively. The sequence for the primer used for the Sanger sequencing was 5′- TTTTAGGAAGCCTACGCCGT-3′.

### Postmortem examination

The diagnosis for the deceased patient (F1-IV-2) in Family 1 was confirmed pathologically by a postmortem examination. Immediately after the postmortem examination, organs were fixed in 10% formalin, and 7 µm-thick paraffin-embedded sections were stained with haematoxylin and eosin and Klüver–Barrera staining. Immunohistochemistry was performed with rabbit polyclonal antibody against COL4A1 (1:100, SAB4300825, Sigma-Aldrich, St Louis, MO) and α-smooth muscle actin (1:250, ab7817, Abcam, Cambridge, MA) through the use of a Ventana BenchMark GX automated slide staining system (Ventana Medical Systems, Tucson, AZ) in accordance with the instructions of the manufacturer.

### Statistical analysis

We calculated a positive predictive value for the radiological feature shared among the Family 1 patients during the cohort screening. Fisher’s exact test was performed by using R for assessing the correlation between the shared radiological features and clinical features. A value of *P* < 0.05 was considered significant.

## Results

### Clinical features

**Figure 1:**
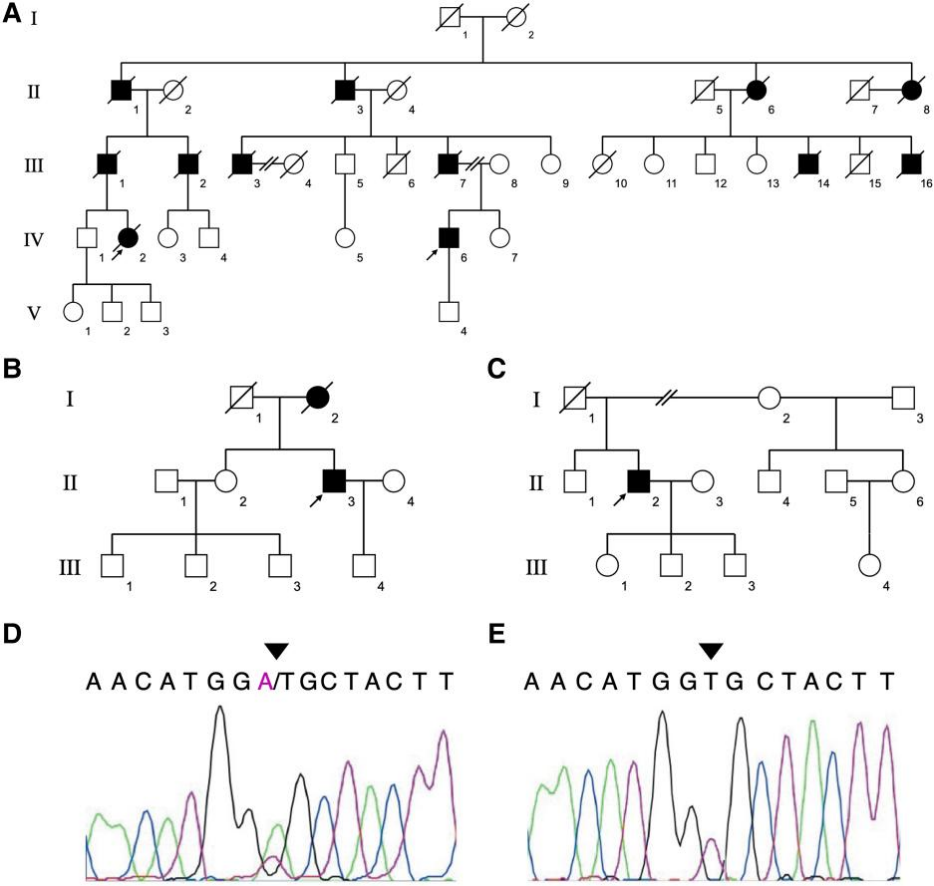
**The pedigree charts of three PADMAL families (Family 1–3) and the identified variant in *COL4A1* 3′-untranslated region (UTR).** (**A**–**C**) Square symbols indicate men and round symbols indicate women. Closed symbols indicate affected individuals proven by clinical histories or MRI. Diagonal lines indicate deceased individuals. Open symbols indicate individuals without PADMAL proven clinically or radiologically. Arrows indicate probands. The pedigree charts of F1 (**A**) and F2 (**B**) show autosomal dominant inheritance pattern while F3 (**C**) does not. (**D**) Sanger sequencing was performed with the proband (F1-IV-2) in F1. The heterozygous variant of *COL4A1* located in c.*33T > A was identified, which is indicated by an arrowhead. (**E**) The healthy control in F1 (F1-IV-1) showed no variant in *COL4A1* 3′UTR in Sanger sequencing.

A pedigree chart of Family 1 is depicted in Fig. 1A, while Supplementary Table 1 presents the clinical features of the two examined patients (F1-IV-2 and F1-IV-6).

**Figure 2:**
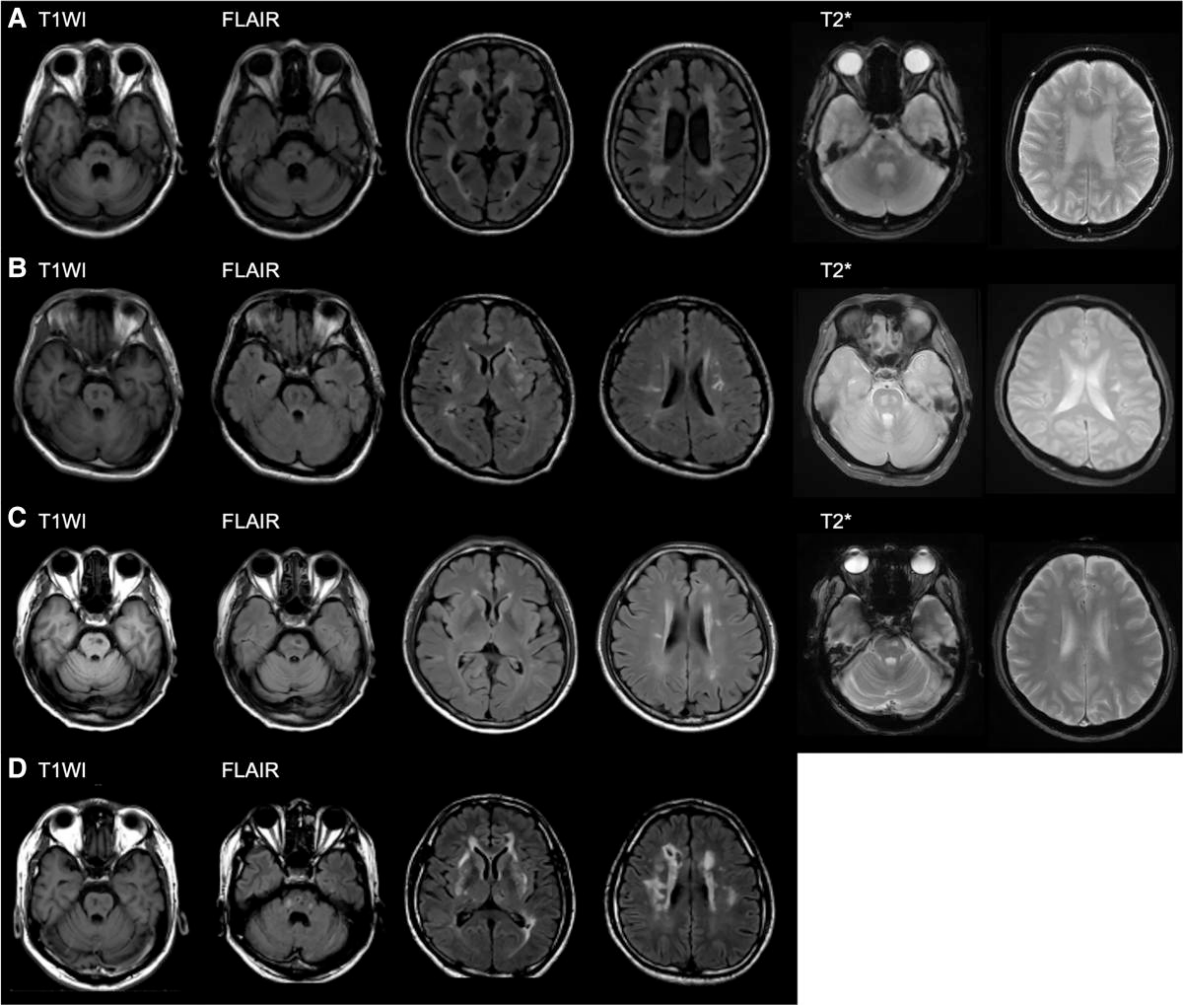
**MRI features of the probands in Family 1–3.** (**A**–**D**) MRI scan was performed using the genetically proven probands at 1.5 T in Family 1 and at 3 T in Family 2 and 3. The examination was performed 9 years after the onset for F1-IV-2 (**A**), 7 years after the onset for F1-IV-6 (**B**), 8 years after the onset for F2-II-3 (**C**) and 12 years after the onset for F3-II-2 (**D**). T1-weighted images (T1WI) show low intensity areas located in the pons and white matter in all four cases. The pontine atrophy was observed in the autopsy case (F1-IV-2). Multiple infarctions were observed in the pons on fluid attenuated inversion recovery and corresponded to the low intensity areas depicted on T1WI. Every patient showed at least one oval shape lesion in the pons. Bilateral white matter hyperintensity was also observed in all cases.

The first patient in Family 1 was F1-IV-2. She had hemiparesis on her right lower extremity at the age of 39 and was diagnosed with a brain infarction without any vascular risk factors. After the first episode, she had suffered from recurrent brain infarctions despite continuous administration of antiplatelets, and stepwise cognitive impairment was observed at 45 years of age. Her brain MRI showed bilateral diffuse ischaemic lesions and bilateral white matter hyperintensities (Fig. 2A). Her paresis gradually worsened, leading to a bedridden state before the age of 57 years (Supplementary Table 1). At the age of 57, her brain MRI showed bilateral cerebellar infarctions (Supplementary Fig. 3). The patient passed away at 59 years of age. The postmortem examination was performed at 13.5 h after her death.

The second patient in Family 1 was F1-IV-6. At the age of 35, he had sudden hemiparesis and anaesthesia in his right extremities and dysarthria, and MRI showed a brain infarction (Fig. 2B). He also suffered from recurrent infarctions despite continuous administration of antiplatelets. His MRI findings and intolerance of antiplatelets suggested that he could suffer from multiple sclerosis. However, the examination of cerebrospinal fluid and the evaluation by contrast-enhanced MRI did not show any findings suggesting multiple sclerosis. While he showed only slight hemiparesis, deep tendon reflex in his extremities was exaggerated asymmetrically. Moreover, Chaddock reflex was present in his left foot suggesting a pyramidal sign.

F1-III-7, the father of F1-IV-6, had shown euphoria with a history of recurrent brain infarctions and died at the age of 62. Bilateral hyperreflexia and pyramidal sign were the shared clinical features found among the patients in Family 1.

### Genetic analysis

We searched for the variants shared by F1-IV-2 and F1-IV-6 in whole exome sequencing and detected six heterozygous variants. The shared variants included two intronic deletions in *GCM2*, one exonic single nucleotide variant in *HECTD2*, one single nucleotide variant in *COL4A1* 3′UTR, one intronic single nucleotide variant in *COL4A2* and one single nucleotide variant in *ING1* 5′UTR. Among these genes, *COL4A1* 3′UTR was the only site reported to be causative of adult cSVD. All of the other variants were either benign or not related to adult cSVD based on ClinVar (ncbi.nlm.nih.gov/clinvar/) and OMIM (ncbi.nlm.nih.gov/omim). The identified variant, *COL4A1*:c.*33T > A, with a combined annotation dependent depletion score of 13.85, was identical to the variant reported in the previous study of cSVD.^10^ The variants in *COL4A1* 3′UTR have been reported to be causative of PADMAL,^6^ and patients F1-IV-2 and F1-IV-6 were diagnosed with PADMAL. The same single nucleotide variant in *COL4A1* 3′UTR was absent in F1-IV-1.

On the basis of the whole exome sequencing results, we confirmed the variant in the 3′UTR of *COL4A1* by Sanger sequencing using the genomic DNA of F1-IV-2 and F1-IV-6. We detected heterozygous *COL4A1*:c.*33T > A in these patients, while the variant was absent in the controls (Fig. 1D and E, Supplementary Fig. 1A).

### Radiological features

F1-IV-2 and F1-IV-6 were examined by 1.5 T MRI and had multiple small infarctions in both the pons and the subcortical hemispheric area (Fig. 2A and B). Fluid attenuated inversion recovery showed the presence of white matter hyperintensities in both patients, which were more broad and severe in F1-IV-2. No severe hemispheric microbleeds were seen by gradient echo T2 (T2*)-weighted images in both patients. MR angiography showed that there was no severe stenosis in depicted vessels including the anterior, middle and posterior cerebral arteries, internal carotid arteries, basilar artery and vertebral arteries in either of the patients (Supplementary Fig. 2A and B). The multiple small oval lesions in the pons were especially characteristic and shared by F1-IV-2 and F1-IV-6, while this feature was absent in F1-IV-1 (control). As the appearance of the pons that contained these characteristic small oval infarctions resembled raisin bread, we coined the name ‘raisin bread sign’ for this unique radiological feature. The raisin bread sign was defined as containing at least two or more oval ischaemic lesions, with distribution on the bilateral sides of the pons, except at the edges.

### Postmortem pathological findings

We evaluated the clinicoradiological feature of one PADMAL patient (F1-IV-2) pathologically during a postmortem examination.

**Figure 3:**
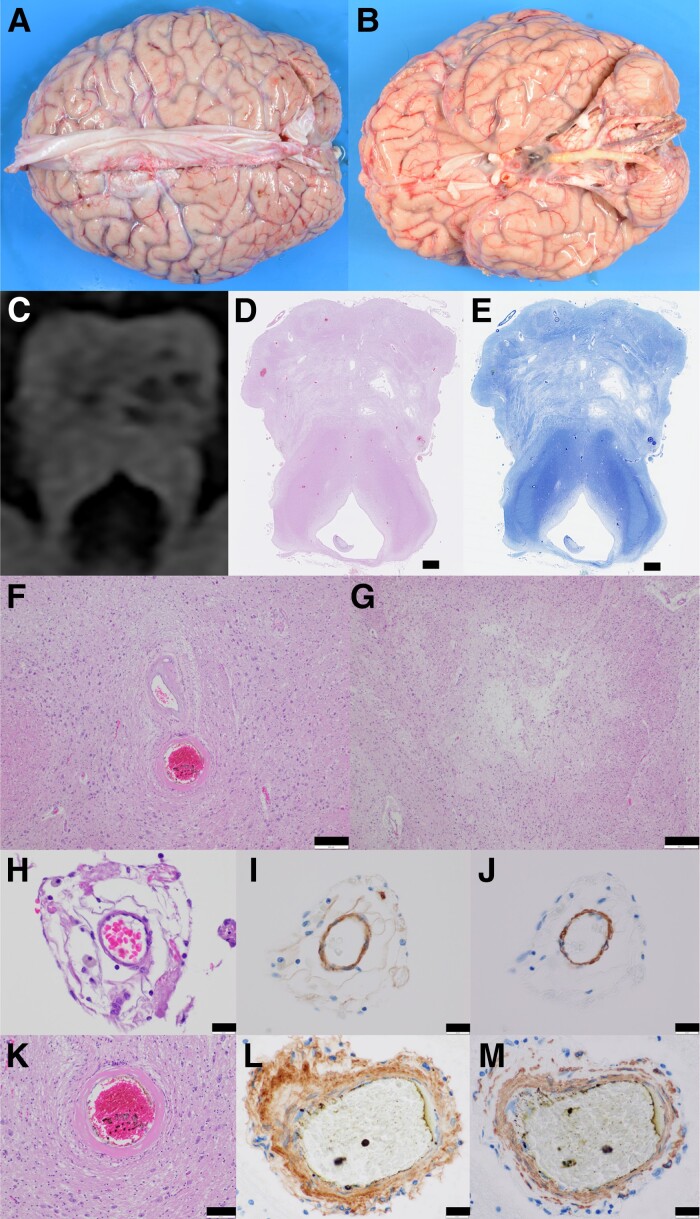
**Histopathology in F1-IV-3.** (**A**, **B**) Gross finding of the brain of F1-IV-2. Atrophies of frontotemporal lobes and brainstem, and the atherosclerosis of the basilar artery were observed. (**C**) The focused image of the pons on fluid attenuated inversion recovery. (**D, E**) Haematoxylin and eosin staining (**D**) and Klüver–Barrera staining (**E**) sections of the pons showed multiple infarctions and existing lesions revealed by MRI. (**F**) Hyalinized vessels in the non-ischaemic lesions had thickened media tunica. (**G**) The pontine ischaemic lesions did not include vessels with thick media tunica. (**H–J**) Vessels in ischaemic lesions (**H**) showed immunopositivity of COL4A1 (**I**) and α-smooth muscle actin (**J**) around the vessels. (**K–M**) Vessels in the non-ischaemic areas (**K**) was hyalinized with thick media tunica, which were immunopositive for COL4A1 (**L**) and α-smooth muscle actin (**M**). Scale bars: (**D, E**) 1 mm, (**F, G**) 200 μm, (**H–J, L, M**) 20 μm, (**K**) 100 μm.

Brain weight was 1160 g and the gross examination revealed atrophy of brainstem, and atherosclerosis of the basilar artery (Fig. 3A and B). Oval ischaemic lesions in the pons were observed at the locations identical to that of the raisin bread sign observed on the MRI (Fig. 3C–E). Histopathological examination revealed hyalinized vessels and thickened media tunica of arterioles in the tissue spared from the ischaemia (Fig. 3F), while the ischaemic lesions did not show these features (Fig. 3G and H). Media tunica of arterioles, regardless of the presence of ischaemic lesions, was positive for COL4A1 (Fig. 3I and L) and α-smooth muscle actin (Fig. 3J and M) on immunohistochemistry, with accumulated COL4A1 only recognized in the thickened media tunica (Fig. 3K and L). Histopathological observation also revealed pyramidal tract atrophy in the pons, medulla oblongata and cervical spinal cord, including the corticospinal tract (Fig. 3D and E).

### Screening for the raisin bread sign

Subsequently, we then screened the cohort of juvenile CVD patients at Hiroshima University Hospital for clinicoradiological features and selected patients found to have the raisin bread sign. Supplementary Table 2 presents the population characteristics of the cohort including the settings of radiological evaluation performed with these patients. In this cohort, four patients were evaluated only by CT and exhibited obvious infarctions in the cortex or basal ganglia without lesions in the pons. Three of them were diagnosed with ‘cardio-embolism’ and had cardiac devices. The other one had non-bacterial thrombotic endocarditis caused by endometrial cancer and was diagnosed with ‘cancer-associated stroke’. From this cohort, we identified two patients showing the raisin bread sign and stroke subtypes of both of them were classified as ‘others undetermined’. Although one CVD patient (F2-II-3) had a familial history of young-onset brain infarction, the other CVD patient (F3-II-2) did not. Figure 1 presents their pedigree charts. These patients also had characteristic small oval infarctions in the pons, and multiple small infarctions in the subcortical hemispheric area and white matter hyperintensities (Fig. 2C and D). Microbleeds and stenosis were not observed among these patients (Supplementary Fig. 2C and D). *COL4A1*:c.*33T > A was detected by Sanger sequencing using the genomic DNA of F2-II-3 and F3-II-2 (Supplementary Fig. 1B and C). Based on these results and clinicoradiological features, both patients were diagnosed with PADMAL. Therefore, these results suggest that the raisin bread sign has a high positive predictive value in detecting PADMAL. We also evaluated several clinical signs among patients with and without the raisin bread sign (Supplementary Table 3). Bilateral hyperreflexia was only observed in the group with the raisin bread sign.

## Discussion

Previous studies have not precisely evaluated the diagnostic value of radiological features of PADMAL, with genetic analysis the only method used to diagnose PADMAL. Early diagnosis, however, is beneficial in predicting the prognosis and preventing excess administration of antiplatelets. In this study, by analysing a family with *COL4A1* 3′UTR variants, we were able to determine that the pontine multiple small oval infarctions were a characteristic radiological feature of patients with PADMAL, with this radiological feature referred to as the raisin bread sign. We also were able to verify this radiological feature of PADMAL during a postmortem pathological examination. Subsequently, we then identified two patients among a cohort of young stroke patients who exhibited the raisin bread sign on MRI. Further evaluation confirmed that they harboured the heterozygous variants in *COL4A1* 3′UTR. The screening results for our juvenile CVD cohort demonstrated that this radiological feature was useful for the genetic diagnosis of PADMAL.

As described in a previous study, although PADMAL is radiologically characterized by pontine involvement with multiple infarcts, the detailed characteristics and specific shapes of the ischaemic lesions have remained unclear.^6,11,13,14^ Based on our current radiological analysis, we coined the name ‘raisin bread sign’ for these pontine multiple small oval infarctions that were commonly observed among the patients in Family 1. During the postmortem examination of F1-IV-2, these small infarcts were pathologically confirmed in the pons, reflecting the distribution and shapes depicted as the raisin bread sign on MRI. The causative variant of PADMAL in the 3′UTR of *COL4A1* results in the accumulation of collagen type IV, which leads to fibrosis of the subendothelial space and lamina muscularis in the cerebral small arteries.^6,8^ These pathological changes have been assumed to cause multiple infarctions, and as mentioned previously, thickened tunica media was observed in the postmortem examination in this study.^6,11,15^ Interestingly, hyalinized vessels and thickened tunica media of arterioles were restricted in the area spared from ischaemia. The infarctions were associated with small arteries that had tunica media of normal thickness, and there were no hyalinized vessels within the ischaemic lesions. The distribution of hyalinized vessels was not consistent with that of the lacunar infarctions.^16,17^ Although thickened arteriolar walls are also observed in ischaemic lesions in cerebral autosomal dominant arteriopathy with subcortical infarcts and leukoencephalopathy,^17-19^ the distribution of the small arteries with thickened media tunica in PADMAL was quite different from that observed in cerebral autosomal dominant arteriopathy with subcortical infarcts and leukoencephalopathy. Furthermore, the pyramidal tract atrophy was obvious and intriguingly correlated with the clinical pyramidal signs observed in all patients. In this study, patients with PADMAL started to show the pyramidal sign during the early stages, and this clinical feature reflecting a pathological change may also be helpful in detecting patients with PADMAL among cSVD patients.

Although white matter hyperintensities was also observed among the patients with PADMAL, similar to the other adult-onset genetic leukoencephalopathies,^20^ we tried to establish a radiological sign that was more specific to PADMAL. On the basis of the correlation between the raisin bread sign and the pathological findings that were confirmed in the postmortem examination, we attempted to use this radiological sign during the screening of PADMAL patients that were evaluated among young patients in a CVD cohort. As well as finding involvement of the anterior temporal pole on MRI in cerebral autosomal dominant arteriopathy with subcortical infarcts and leukoencephalopathy that helps facilitate the genetic diagnosis,^3-5^ the results of our study also suggest that the raisin bread sign is an indicative sign of PADMAL. Previous studies also reported pontine MRI findings from 10 genetically confirmed PADMAL patients.^6,11,13^ All their MRI findings presented several oval lesions in pons, which we considered as the raisin bread sign. Thus, the raisin bread sign allows more efficient selection of the candidates for genetic analysis of *COL4A1* 3′UTR. This sign is also effective even when the patient is sporadic, indicating that an absence of family history is not always sufficient for excluding the possibility of PADMAL. One of the limitations of our study was that we did not perform genetic analysis among patients without the raisin bread sign, and thus, precise estimation of the sensitivity and specificity was difficult. In addition, while the patients examined in this study all harboured the same variant in *COL4A1* 3′UTR, the number of multiple infarctions and severity of leukoencephalopathy varied among these patients and was not correlated with the age at the first stroke episode. Therefore, there may be another factor affecting the burden of lesions of PADMAL besides the location of variants.

In a previous report, a variant in a location other than *COL4A1* 3′UTR was identified to be causative of hereditary multi-infarct dementia of the Swedish type, which was characterized by cognitive impairment, depressive illness and behavioural symptoms, including mood changes.^21,22^ These symptoms can also be observed among patients with PADMAL,^6^ with two of our patients exhibiting cognitive dysfunction and mood disturbance as the disease progressed. The phenotypes related to variants in *COL4A1* 3′UTR may be varied as well as those caused by the variants within the coding region of *COL4A1*.^9^

In the present study, we detected an important radiological sign of PADMAL, which we refer to as the raisin bread sign, that can be used as a factor in helping decide on whether to perform genetic analysis for *COL4A1* 3′UTR in patients whose family histories are unavailable. Moreover, the correlation between radiological and neuropathological features recognized in our study could potentially provide important information that can be used in the detection of the detailed mechanism of PADMAL in the future.

## Supplementary Material

fcad281_Supplementary_Data

## Data Availability

Protocols and other non-individual information are available on request.
