# Supplementary material for: ‘Raisin bread sign’ feature of pontine autosomal dominant microangiopathy and leukoencephalopathy
Source: Brain Commun. 2023 Oct 22;5(6):fcad281. doi: 10.1093/braincomms/fcad281 (PMC10636559; doi:10.1093/braincomms/fcad281)

Supplementary Figure 1

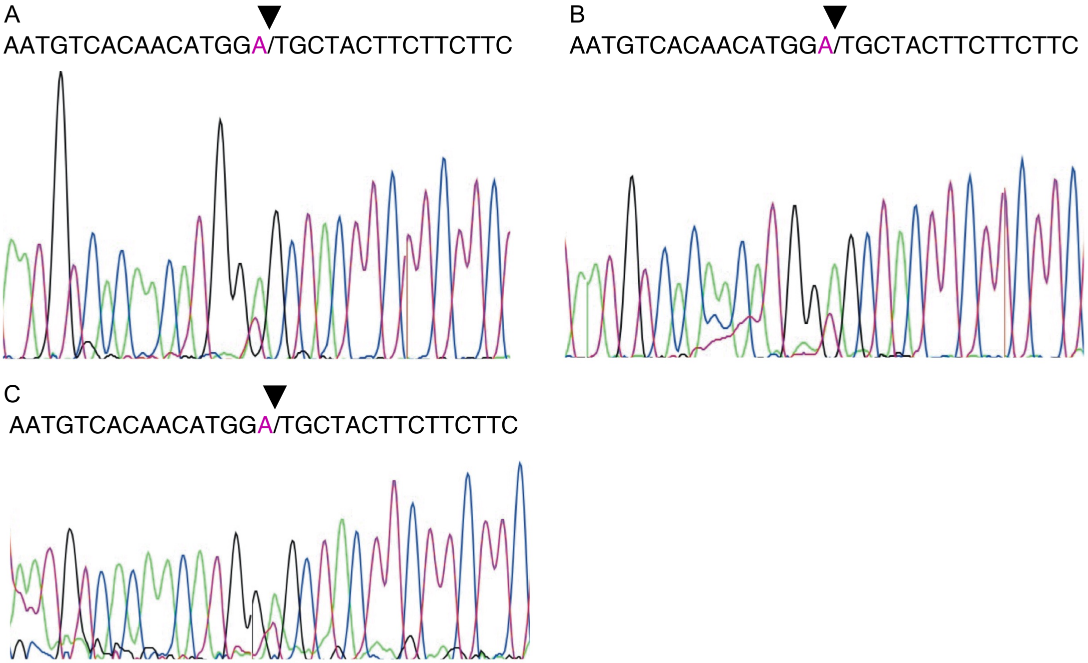

Supplementary Figure 2

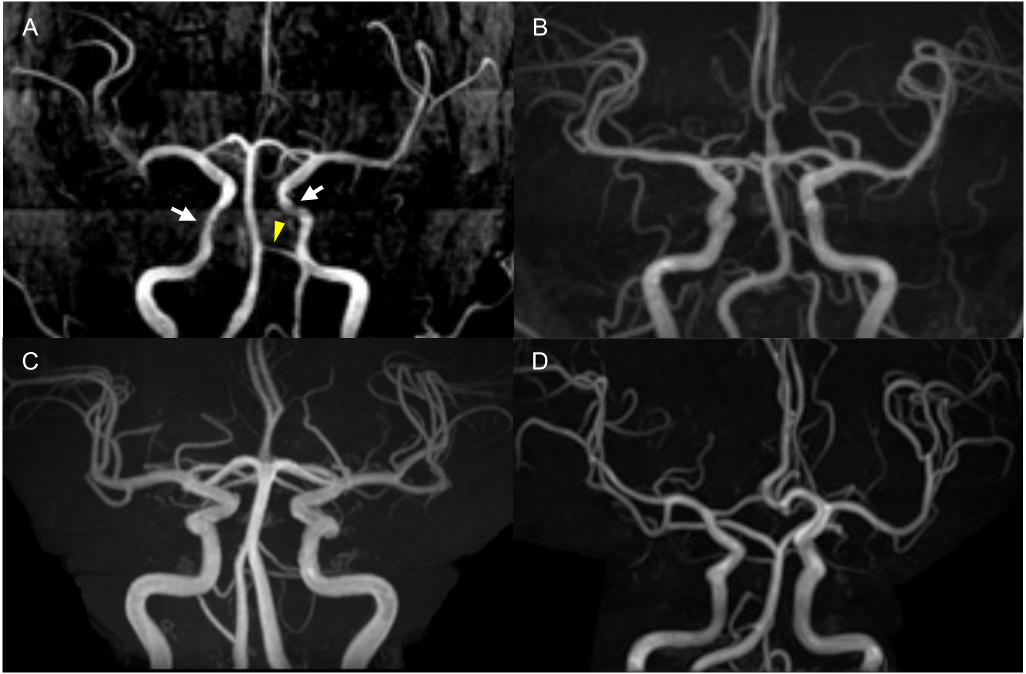

Supplementary Figure 3

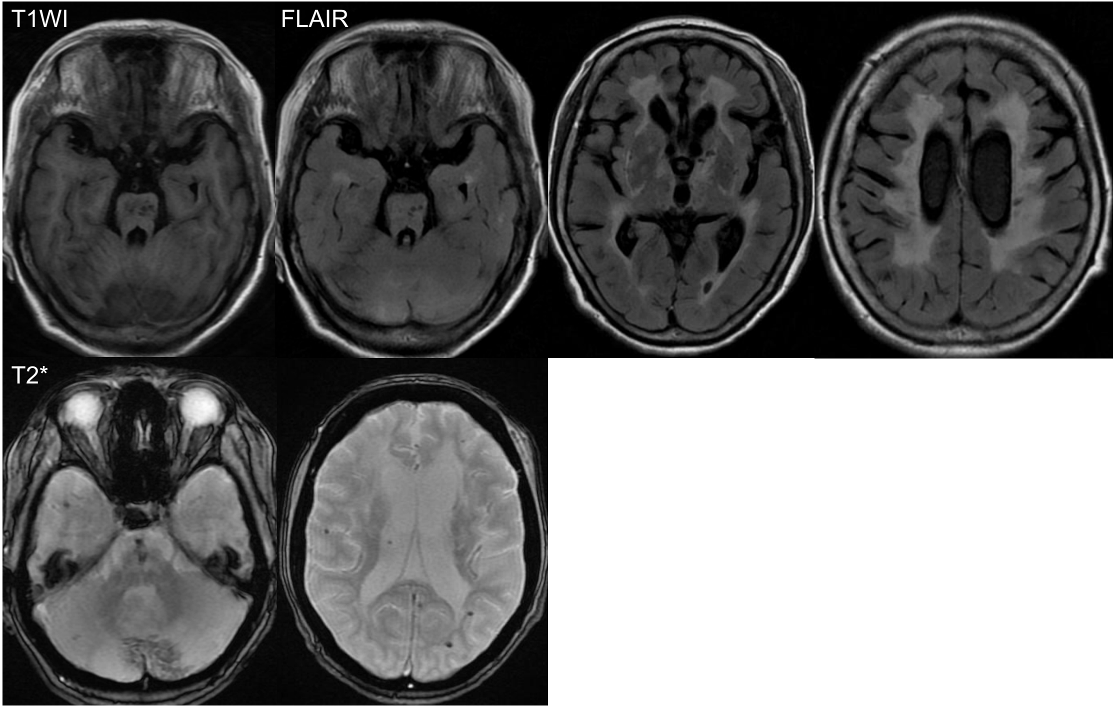

Supplement: fcad281_Supplementary_Data [file fcad281_supplementary_data.zip › Supplementary_Figures.pdf]
