# Supplementary material for: ‘Raisin bread sign’ feature of pontine autosomal dominant microangiopathy and leukoencephalopathy
Source: Brain Commun. 2023 Oct 22;5(6):fcad281. doi: 10.1093/braincomms/fcad281 (PMC10636559; doi:10.1093/braincomms/fcad281)
Supplement: fcad281_Supplementary_Data [file fcad281_supplementary_data.zip › Supplementary_Tables.docx]

**Supplementary Table 1 Clinical features of the patients with PADMAL**

| **Patient** | **Age at 1st stroke**  **(y)** | **mRS**  **(age, y)** | **Paresis** | **Bilateral hyperreflexia** | **Foot clonus** | **Cognitive impairment and/or mood disturbance** |
| --- | --- | --- | --- | --- | --- | --- |
| F1-IV-2 | 39 | 5 (57) | Hemiparesis in right extremities | + | − | + |
| F1-IV-6 | 35 | 1 (41) | Mild paresis in left plantar flexor and dorsiflexor (MMT 4) | + | + | − |
| F2-II-3 | 40 | 2 (48) | Mild paresis in right extremities (Barré’s sign and Mingazzini sign are positive.) | + | +  (pseudo-clonus) | − |
| F3-II-2 | 36 | 3(49) | Mild paresis in left upper extremity (Barré’s sign is positive.) | + | +  (pseudo-clonus) | + |

mRS, modified Rankin scale; MMT, manual muscle testing

**Supplementary Table 2 Characteristics of the cohort of juvenile CVD patients**

| Number of the patients | 40 |
| --- | --- |
| Age at onset, mean, y (SD) | 43.3 (5.3) |
| Female, n (%) | 9 (23.1) |
| Stroke subtype |  |
| Large artery atherosclerosis, n (%) | 3 (7.7) |
| Small vessel occlusion, n (%) | 2 (5.1) |
| Cardio-embolism, n (%) | 5 (12.8) |
| Cancer-associated stroke, n (%) | 4 (10.3) |
| Cerebral artery dissection, n (%) | 9 (23.1) |
| Others undetermined, n (%) | 17 (43.6) |
| Radiological evaluation |  |
| 3T MRI, n (%) | 32 (80) |
| 1.5T MRI, n (%) | 16 (40) |
| CT-only, n (%) | 4 (10) |
| Patients with pontine lesions, n (%) | 8 (20.5) |
| Raisin bread sign, n (%) | 2 (5.1) |

CVD, cerebral vessel disease; SD, standard deviation; CT, computed tomography

**Supplementary Table 3 Clinical features of juvenile CVD patients with or without raisin bread sign**

|  | **Raisin bread sign positive (n = 2)** | **Raisin bread sign negative (n = 38)** |  |
| --- | --- | --- | --- |
| Hyperreflexia | 2 | 6 | *p* = 0.1014 |
| Bilateral hyperreflexia | 2 | 0 | *p* = 0.003623 |
| Paresis | 2 | 17 | *p* = 0.2192 |
| Mild paresis  (Barré’s sign, Mingazzini sign  or Wartenberg’s sign with MMT 4–5) | 2 | 5 | *p* = 0.05172 |
| Cognitive impairment  or higher brain dysfunction | 1 | 10 | *p* = 0.4899 |
| Mood disturbance | 1 | 8 | *p* = 0.4038 |

MMT, manual muscle testing
