# Supplementary material for: ‘Raisin bread sign’ feature of pontine autosomal dominant microangiopathy and leukoencephalopathy
Source: Brain Commun. 2023 Oct 22;5(6):fcad281. doi: 10.1093/braincomms/fcad281 (PMC10636559; doi:10.1093/braincomms/fcad281)
Supplement: fcad281_Supplementary_Data [file fcad281_supplementary_data.zip › Supplementary_Figure_Legends.docx]

**Supplementary Fig. 1 Results of Sanger sequencing**

(**A–C**) Result of the Sanger sequencing performed in F1-IV-6 (**A**), F2-II-3 (**B**), and F3-II-2 (**C**) showed the heterozygous variant of collagen type IV alpha chain (*COL4A1*) located in c.*33T>A, which was identical to F1-IV-2.

**Supplementary Fig. 2 Magnetic resonance angiography**

(**A**) Magnetic resonance angiography (MRA) showed slight stenosis in both carotid arteries (arrows) and the left vertebral artery (arrowhead) in F1-IV-2. (**B–D**) No stenosis was observed in F1-IV-6, F2-II-3 and F3-II-2 on MRA.

**Supplementary Fig. 3** **MRI performed during the early period in F1-IV-2**

MRI was performed at 17 years after F1-IV-2 onset. Multiple bilateral infarctions and WMH were observed in the pons on FLAIR. Although low intensity areas were observed on T1WI that corresponded to the multiple infarctions shown on FLAIR, pontine atrophy was not obvious.
